# Simulation training with haptic feedback of instrument vibrations reduces resident workload during live robot-assisted sleeve gastrectomy

**DOI:** 10.1007/s00464-024-11459-6

**Published:** 2024-12-31

**Authors:** Ernest D. Gomez, Haliza Mat Husin, Kristoffel R. Dumon, Noel N. Williams, Katherine J. Kuchenbecker

**Affiliations:** 1 Department of Otolaryngology – Head and Neck Surgery, Harvard Medical School, Boston, MA USA; 2 Department of Mechanical Engineering and Applied Mechanics, University of Pennsylvania, Philadelphia, USA; 3 Haptic Intelligence Department, Max Planck Institute for Intelligent Systems, Heisenbergstraße 3, 70569 Stuttgart, Germany; 4 Department of Surgery, Hospital of the University of Pennsylvania, Philadelphia, USA; 5 Perelman School of Medicine, University of Pennsylvania, Philadelphia, USA; 6 Penn Medicine Clinical Simulation Center, Penn Medicine Rittenhouse, Philadelphia, USA

**Keywords:** Haptic feedback, Naturalistic vibrotactile feedback, Robotic surgery, Surgical simulation, Subjective workload assessment, NASA-TLX

## Abstract

**Background:**

New surgeons experience heavy workload during robot-assisted surgery partially because they must use vision to compensate for the lack of haptic feedback. We hypothesize that providing realistic haptic feedback during dry-lab simulation training may accelerate learning and reduce workload during subsequent surgery on patients.

**Methods:**

We conducted a single-blinded study with 12 general surgery residents (third and seventh post-graduate year, PGY) randomized into haptic and control groups. Participants performed five simulated bariatric surgeries on a custom inanimate simulator followed by live robot-assisted sleeve gastrectomies (RASGs) using da Vinci robots. The haptic group received naturalistic haptic feedback of instrument vibrations during their first four simulated procedures. Participants completed pre-/post-procedure STAI and post-procedure NASA-TLX questionnaires in both simulation and the operating room (OR).

**Results:**

Higher PGY level (simulation: *p* < 0.001, OR *p* = 0.004), shorter operative time (simulation: *p* < 0.001, OR *p* = 0.003), and lower pre-procedure STAI (simulation: *p* = 0.003, OR *p* < 0.001) were significantly associated with lower self-reported overall workload in both operative settings; PGY-7 s reported about 10% lower workload than PGY-3 s. The haptic group had significantly lower overall covariate-adjusted NASA-TLX during the fourth (*p* = 0.03) and fifth (*p* = 0.04) simulated procedures and across all OR procedures (*p* = 0.047), though not for only the first three OR procedures. Haptic feedback reduced physical demand (simulation: *p* < 0.001, OR *p* = 0.001) and increased perceived performance (simulation: *p* = 0.031, OR *p* < 0.001) in both settings.

**Conclusion:**

Haptic feedback of instrument vibrations provided during robotic surgical simulation reduces trainee workload during both simulation and live OR cases. The implications of workload reduction and its potential effects on patient safety warrant further investigation.

Studies on robot-assisted surgery (RAS) have suggested that the technology may offer several advantages over open surgery and conventional laparoscopic surgery including reduced blood loss, reduced postoperative pain, reduced risk of infection, and shorter recovery time for patients undergoing select surgical procedures [1, 2]. These potential benefits complement other suggested benefits of RAS such as improved visualization, enhanced dexterity, tremor attenuation, and favorable ergonomics for surgeons when compared to “straight stick” laparoscopic surgery [2–4]. However, the lack of haptic (touch) feedback in widespread commercial robotic surgical systems imposes perceptual-motor challenges for users [5, 6], especially when manual palpation and sensation of tension are important for the task, such as during knot tying and soft tissue dissection.

Acquisition of novel skills and adoption of new technologies can pose mental and physical challenges for all surgeons, especially trainees. Cognitive workload is described as the ratio between resource demand and total resources available [7]. Previous research has indicated that cognitive overload in complex events or emergencies could impair surgical performance [8, 9]. Higher surgeon fatigue and mental demand during surgery may lengthen procedures and increase error rates [10–12], thus risking clinical performance and eventually patient care.

In RAS, visual feedback is the dominant source of sensory input available to the surgeon, and the sense of touch is essentially eliminated when compared to both open and laparoscopic surgery. Operating under such sensory conditions may elevate workload and can increase the risk of surgical errors, especially among trainees. Even though studies suggest that robotic surgery can reduce workload compared to manual laparoscopic surgery [5, 13–16], studies of robotic surgery have demonstrated poorer performance in terms of decreased accuracy and slower completion of simulated surgical tasks [17].

Haptic feedback is omnipresent in open surgery, and its value has been best elucidated for practitioners by its gradual loss as laparoscopic and robotic surgery were adopted. Many studies have demonstrated a value for haptic feedback in robotic surgery [18, 19]. For instance, various forms of haptic feedback have been found to positively impact teleoperated task performance by decreasing the number of errors [20], decreasing tissue damage [21], lowering contact forces exerted on tissue [22], causing the operator to move more cautiously [23], and improving the realism of physical interactions [24, 25]. Furthermore, learning efficiency increased in the early surgical training stage when performing laparoscopic suturing and knot-tying tasks with haptic feedback [26].

The direct clinical advantage of haptic feedback is challenging to investigate during live robotic surgery due to the regulatory processes for approval and modification of surgical devices. Thus, investigations into the effects of haptic feedback on surgical performance have predominantly taken place during surgical simulation. Such research studies have generally demonstrated the added value of haptic feedback in improving both learning and technical skill. However, less is known about how haptic feedback experienced by learners in the setting of simulation would affect the subsequent performance of real robotic surgery, which is almost universally performed without haptic feedback.

In a virtual surgical training environment using the ProMIS laparoscopic simulator, surgeons who were cognitively loaded performed a training task more accurately when force feedback was provided [27]. However, providing force feedback safely during live robotic surgery is technically challenging [28]. McMahan et al. [29] implemented a haptic feedback system called VerroTouch that uses low-cost sensors and actuators to allow the surgeon to feel the vibrations of the left and right da Vinci instruments at their fingertips; such vibrations are caused by sudden contact with a stiff object, releases of tension, and other transient physical events. A user study evaluating VerroTouch found that providing this naturalistic vibrotactile feedback did not interfere with teleoperation and positively affected the surgeon’s perceived concentration ability. In vivo testing of VerroTouch on a porcine model demonstrated detection and presentation of vibration transients for 82% of surgeon actions [30]. In a follow-on study that compared both haptic and auditory feedback of these vibrations to a standard da Vinci robot, the haptic feedback was found to increase the operator’s awareness of instrument contact interactions during training tasks, and its addition was strongly preferred by both surgeons and non-surgeons [31]. Another investigation showed that measured instrument vibrations are a construct-valid indicator of surgical skill, with experienced surgeons causing smaller vibrations than novices in certain simulation-based tasks [32]. Furthermore, the presence of naturalistic vibrotactile feedback allowed one experienced surgeon to execute in vitro surgical training tasks with lower contact forces while maintaining fast completion times [33]. These results suggest that providing this type of haptic feedback could support learning and surgical task performance during training.

This current study aims to understand how naturalistic haptic feedback of instrument vibrations affects user experience during early learning of robotic surgery. By providing haptic feedback during dry-lab simulations of robotic bariatric surgery and assessing the subjective workload after both simulated and live operating room (OR) procedures, we sought to (1) identify potential demographic and procedural influences on the residents’ workload and (2) characterize the effect of haptic feedback on workload in these settings. This study utilized the VerroTouch system [29, 34] to provide the surgeon with realistic vibrotactile feedback through the da Vinci handles. We administered the National Aeronautics and Space Administration Task Load Index (NASA-TLX) [35] to subjectively measure residents’ workload after every procedure. We expected factors like baseline stress, procedure completion time, and level of experience would influence the residents’ workload. We also hypothesized that residents who trained with haptic feedback would have a lower experienced workload in both simulated and real bariatric surgery.

## Materials and methods

### Study participants

Twelve residents (10 male and 2 female) from the Hospital of the University of Pennsylvania participated in the study. Participants were general surgery residents in either their third or seventh post-graduate year (PGY-3 or PGY-7), and they ranged in age from 29 to 36 years. All participants were right-hand dominant. Data on demographic information, laparoscopic and robotic surgical experience, and medical conditions were recorded. Participant demographics are summarized in Table 1. The Institutional Review Board of the University of Pennsylvania approved the study plan under protocol #815,301, and all participants provided written informed consent prior to participation in the study.

**Table 1:** Demographic information of study participants

Characteristic	Control (*n* = 6)	Haptic (*n* = 6)
Age (years)	31.8 ± 3.0	31.3 ± 2.6
Male: Female	5:1	5:1
Right hand dominance	6/6	5/6
PGY-3: PGY-7	3:3	4:2
Video Game Play > 0 Hours/Week	1/6	0/6
Assisted in 1 robot case in past 7 days	4/6	4/6
Prior laparoscopic case experience	122.2 ± 90.1	121.7 ± 78.6

### Study procedure and design

Participants were randomized to one of two groups: the haptic group received haptic feedback from the VerroTouch device [29] during the first four of five bariatric surgery simulations, and the control group received no haptic feedback during any of the five bariatric surgery simulations. To balance groups by training level, the first participant to consent to the study (a PGY-3 resident) was randomized to the haptic group. Since one PGY-3 and one PGY-7 were on the bariatric surgery service at any given time, the first PGY-7 participant recruited into the study was assigned to the control group. For the next pair of residents, the PGY-7 was assigned to the haptic group, and the PGY-3 was assigned to the control group. All subsequent pairs of residents alternated in this manner. Resident skill level was not a determining factor in the rotation schedule, so this method was presumed to achieve acceptable randomization while balancing groups by training level. To prevent bias, the attending surgeons supervising the trainees during their clinical service were blinded to the group assignments.

**Fig. 1:**
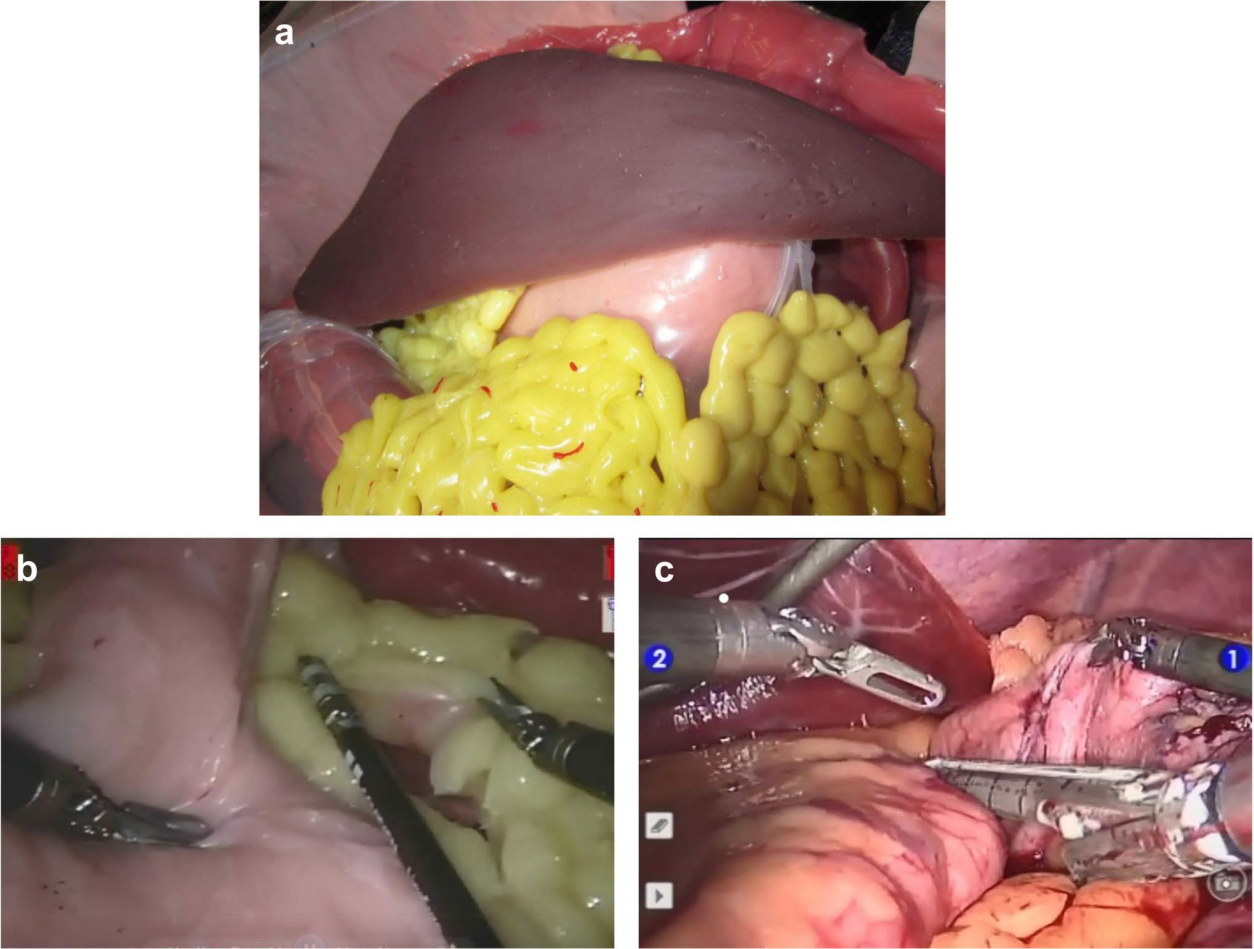
Simulator and robotic bariatric procedures. **a** Custom abdominal simulator used for the simulated procedures, and sample video frames from the **b** simulated and **c** live OR robot-assisted sleeve gastrectomy (RASG) procedures

The simulation procedures used a physical abdominal organ model [36], which included the stomach, spleen, pancreas, and liver, as shown in Fig. 1. Organ models were initially created using clay sculptures that were used to cast reusable silicone molds. The organ model environment was found to have acceptable face and content validity for simulation of robot-assisted gastric banding (RAGB) and robot-assisted sleeve gastrectomy (RASG) procedures.

At the session for the simulation procedures, participants watched an instructional video on RAGB and RASG, followed by 20 min of practice time on a da Vinci Standard surgical robot. Participants used this practice time to adapt to instrument handling, practice camera control and clutching, and perform the common training tasks of peg transfer and intracorporeal suturing. Participants reported their baseline stress levels using a previously validated short form of the State-Trait Anxiety Inventory (STAI) survey [37–39]. Total STAI score was calculated by summing the numerical responses to the six individual questions (calm, tense, upset, relaxed, content, and worried) after reversing the scores of tense, upset, and worried to give all categories the same polarity.

**Fig. 2:**
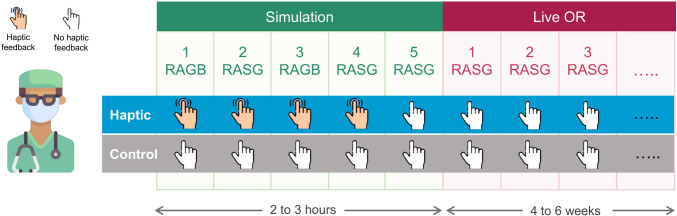
Study flowchart. After participants were randomized to either the haptic group or the control group, they performed five high-fidelity procedure-specific simulations: two trials of robot-assisted gastric banding (RAGB) and three trials of robot-assisted sleeve gastrectomy (RASG). Starting the following day, participants performed live RASG’s on patients for the duration of their gastrointestinal surgery rotation

Participants then performed the first simulated RAGB followed by a simulated RASG procedure, and they repeated the process for three more procedures: a RAGB, a RASG, and one final RASG, as depicted in the study flowchart in Fig. 2. A five-minute break was provided after the completion of each procedure. Haptic feedback was removed for the final simulated RASG procedure to match the sensory conditions participants would encounter in the OR. Completion of these five procedure-specific simulations took between two and three hours. Upon completion of each simulated procedure, participants were again administered the STAI survey to assess pre- and post-task changes in their stress level as well as the six subscales of the NASA-TLX questionnaire [35] to measure their perceived workload. On the day following the simulation session, participants from both groups performed the RASG procedure in a live OR setting on a clinically operational da Vinci Si robot without haptic feedback; due to changes in standard of care clinical practice that occurred during the design and implementation of the study, no live RAGB procedures were performed in the OR during this study. Pre- and post-procedure stress level and post-procedure workload were assessed in the live OR in the same manner as done in the simulation setting (pre-procedure STAI, post-procedure STAI, and post-procedure NASA-TLX). Video from the endoscopic camera and the instrument vibrations were recorded during all procedures using a DVD recorder [34]. Figures 1b and c show samples of video images during simulated and live OR bariatric procedures.

### Workload metrics

Participant workload was examined in two ways: (1) the normalized overall NASA-TLX scores (*NASA-TLX*_*overall*_) and (2) the scores of the six individual NASA-TLX subscales (mental demand, physical demand, temporal demand, performance, effort, and frustration level). *NASA-TLX*_*overall*_ was calculated as the ratio between the sum of the six NASA-TLX subscale scores and the sum of the maximum possible scores. The raw scores of the NASA-TLX subscales were evaluated individually, as researchers have previously suggested using individual NASA-TLX subscales to assess perceived workload more accurately [40, 41]. Low *NASA-TLX*_*overall*_ scores indicate a low (good) workload, as do low subscale scores for mental demand, physical demand, temporal demand, performance, effort, and frustration level. Note that the performance subscale of the NASA-TLX refers to the participant’s self-perceived performance rather than an external evaluation.

### Statistical analysis

Multiple backward linear regression analyses were performed to investigate whether number of hours slept (prior to the simulation or surgery), pre-procedure STAI score, level of surgical experience, operative time, and haptic feedback could predict resident *NASA-TLX*_*overall*_. An independent t test was used to test the association between PGY group (PGY-3 vs. PGY-7) and *NASA-TLX*_*overall*_. A paired t test matched by participant and case number was used to test for differences in *NASA-TLX*_*overall*_ between simulated RAGB and RASG procedures. To evaluate the difference between the feedback groups (haptic and control) on the individual NASA-TLX subscales, we performed analysis of covariance (ANCOVA). A mixed analysis of covariance (mixed ANCOVA) was performed to test the effects of feedback group and procedure number on the *NASA-TLX*_*overall*_. The Bonferroni-Holm correction for multiple tests was performed when determining significant pairwise comparisons.

## Results

### Study population

A total of 12 eligible individuals (Table 1) of the consecutive cohort completed the study. A summary of the simulated and live OR procedures is presented in Table 2. In total, 60 simulated and 76 live cases were recorded during the study. Prior to the study, none of the PGY-7 participants had performed an RASG because Robotic-Assisted Gastric Banding (RAGB) was the robotic bariatric procedure of choice at the time of their PGY-3 rotation. Laparoscopic case experience prior to study participation is displayed in Table 1 and was not found to be significantly different between groups when compared with using a two-sample Wilcoxon rank-sum test (*p* = 0.933). The structure of the general surgery residency was such that bariatric surgeries were performed almost exclusively during their designated bariatric surgery rotation, and there was no laparoscopic sleeve gastrectomy experience reported by the participants from non-bariatric service rotations. Four of the six participants in each group served as a bedside assistant in one RASG prior to start of the study because the bariatric surgery rotation started on varying days of the week, and the study simulation sessions took place only Wednesdays. Study participants who served as bedside assistants prior to study participation did not act as console surgeons during those cases because robot console training and onboarding routinely took place on Wednesdays, the bariatric rotation’s weekly clinic day.

**Table 2:** Summary data of bariatric cases in simulation and the live OR

Variables	Range	Mean	Standard deviation
*Simulation* *RASG and RAGB cases (n* = *60)*			
Sleep (hours)	5–7	5.96	0.81
Case number	1–3	1.80	0.76
Pre-procedure STAI	6–13	8.25	2.46
Post-procedure STAI	6–15	9.29	3.06
Post-procedure *NASA-TLX*_*overall*_	0.26–0.79	0.44	0.12
Operative time (minutes)	13.55–54.58	25.91	9.95
*Live OR* *RASG cases (n* = *76)*			
Sleep (hours)	1–8	5.86	1.18
Case number	1–14	4.89	3.46
Pre-procedure STAI	6–19	9.26	3.98
Post-procedure STAI	6–17	9.24	3.46
Post-procedure *NASA-TLX*_*overall*_	0.17–0.76	0.50	0.16
Operative time (minutes)	26.47–116.60	54.23	18.32

RASG: Robotic-assisted sleeve gastrectomy, RAGB: Robotic-assisted gastric banding, OR Operating room

## Factors contributing to perceived workload

A backward stepwise multiple regression was performed to investigate if number of hours slept, level of experience, case number, operative time, pre-procedure STAI score, and post-procedure STAI score predicted the *NASA-TLX*_*overall*_. Level of experience was coded as 0 for PGY-3 and 1 for PGY-7 residents.

### Simulation setting

**Fig. 3:**
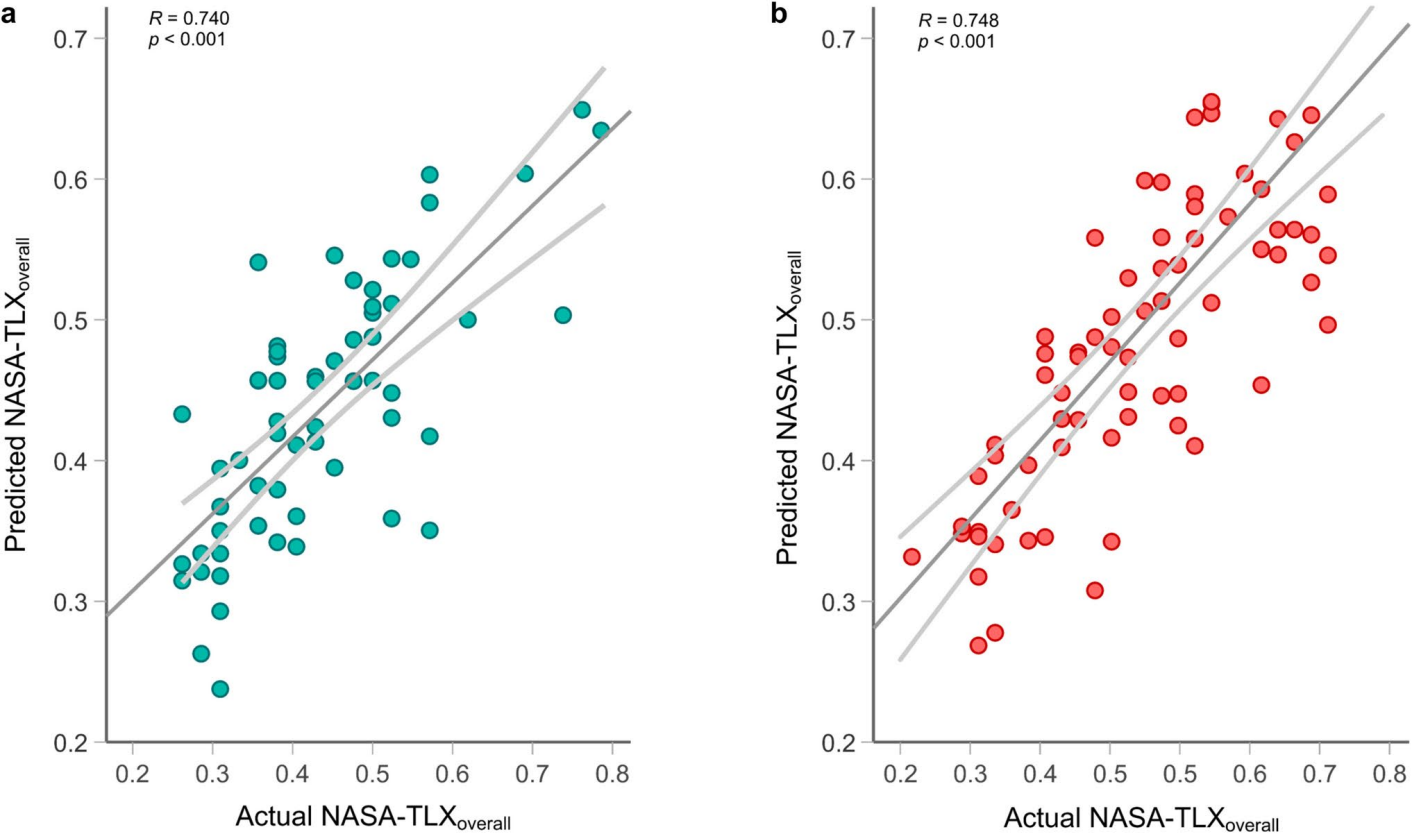
Multiple linear regression for *NASA-TLX*_*overall*_ Correlation between predicted and actual *NASA-TLX*_*overall*_ from **a** simulated and **b** live OR bariatric surgical procedures, with *R* as the correlation coefficient

The regression analysis revealed a collective significant effect of PGY level, sleep, operative time, and pre-procedure STAI score on the *NASA-TLX*_*overall*_ for the simulation setting (F(4, 55) = 16.623, *p* < 0.001, *R*^*2*^ = 0.547, adjusted *R*^*2*^ = 0.514, Fig. 3a, Table 3). These factors could account for approximately 51.4% of the variance in the workload scores. The individual predictors were examined further and indicated that PGY level (*p* < 0.001), sleep level (*p* = 0.002), operative time (*p* < 0.001), and pre-procedure STAI score (*p* = 0.003) were significant predictors in the model. The model for predicting the *NASA-TLX*_*overall*_ during simulated bariatric procedures was

$$ \begin{gathered} NASA - TLX_{{overall\left( {sim} \right)}} = 0.483 - 0.104 \cdot PGY \, Level - \hfill \\ 0.0516{\text{ hr}}^{ - 1} \cdot Sleep + 0.00507\min^{ - 1} + 0.0211 \cdot STAI_{pre} \hfill \\ \end{gathered} $$

**Table 3:** Multiple backward regression for resident workload, *NASA-TLX*_*overall*_

Setting	Parameter	Unstandardized Coefficients		*p*-value
		B	SE	
Simulation *(n* = *60)*	(Intercept)	0.483	0.100	< 0.001
	PGY Level	− 0.104	0.024	< 0.001
	Sleep (hours)	− 0.052	0.016	0.002
	Time (minutes)	0.005	0.001	< 0.001
	Pre-procedure STAI	0.021	0.007	0.003
Live OR *(n* = *76)*	(Intercept)	0.345	0.063	< 0.001
	PGY Level	− 0.096	0.032	0.004
	Case Number	− 0.019	0.004	< 0.001
	Time (minutes)	0.002	0.001	0.003
	Pre-procedure STAI	0.016	0.004	< 0.001

SE: Standard error, OR Operating room, PGY: Post-graduate year, Time: Operative time, STAI: State-Trait Anxiety Inventory

### Live OR setting

For the live OR setting, the multiple regression showed PGY level, total completed cases, operative time, and pre-procedure STAI explained a significant proportion of the variance in *NASA-TLX*_*overall*_ (F(4,71) = 22.603, *p* < 0.001, *R*^*2*^ = 0.560, adjusted *R*^*2*^ = 0.535, Fig. 3b). The model significantly explained 53.5% of the variance in the workload scores. There was a significant contribution of PGY level (*p* = 0.004), case number (*p* < 0.001), operative time (*p* = 0.003), and pre-procedure STAI score (*p* < 0.001) in the model. The regression equation for predicting the *NASA-TLX*_*overall*_ during live OR procedures was

## Effect of PGY level on workload

We assessed the effect of PGY level on the *NASA-TLX*_*overall*_. An independent sample t test revealed a significantly higher average *NASA-TLX*_*overall*_ (higher workload) for PGY-3 residents compared to the more experienced PGY-7 residents in both settings (Simulation: 0.47 ± 0.09 vs. 0.37 ± 0.09, t(32) = 2.96, *p* = 0.006; Live OR 0.50 ± 0.15 vs. 0.36 ± 0.10, t(45) = 2.44, *p* = 0.019).

When analyzing the differences between the feedback groups in both settings, we therefore included PGY level as a covariate to control for its possible effects on the *NASA-TLX*_*overall*_. For the simulation setting, sleep was also added as a covariate since it has a major influence on the *NASA-TLX*_*overall*_, as shown in the multiple linear regression.

## Effect of haptic feedback on workload

### Simulation setting

Data were collected from two procedures, RASG and RAGB. Since each participant performed three RASG and two RAGB procedures, analysis was performed on matched cases from the first two of each kind of procedure. Twenty-four RASG cases and 24 RAGB cases were included. A paired t test matched by participant and case number showed no significant difference in the *NASA-TLX*_*overall*_ of the RASG and RAGB procedures (*p* = 0.075). Based on this result, we combined the data from both procedures (*n* = 60; Haptic = 30, Control = 30) and performed a mixed-model ANCOVA to evaluate the effect of feedback group and procedure number on *NASA-TLX*_*overall*_. PGY level and sleep were used as covariates to control for their influence on the *NASA-TLX*_*overall*_.

**Fig. 4:**
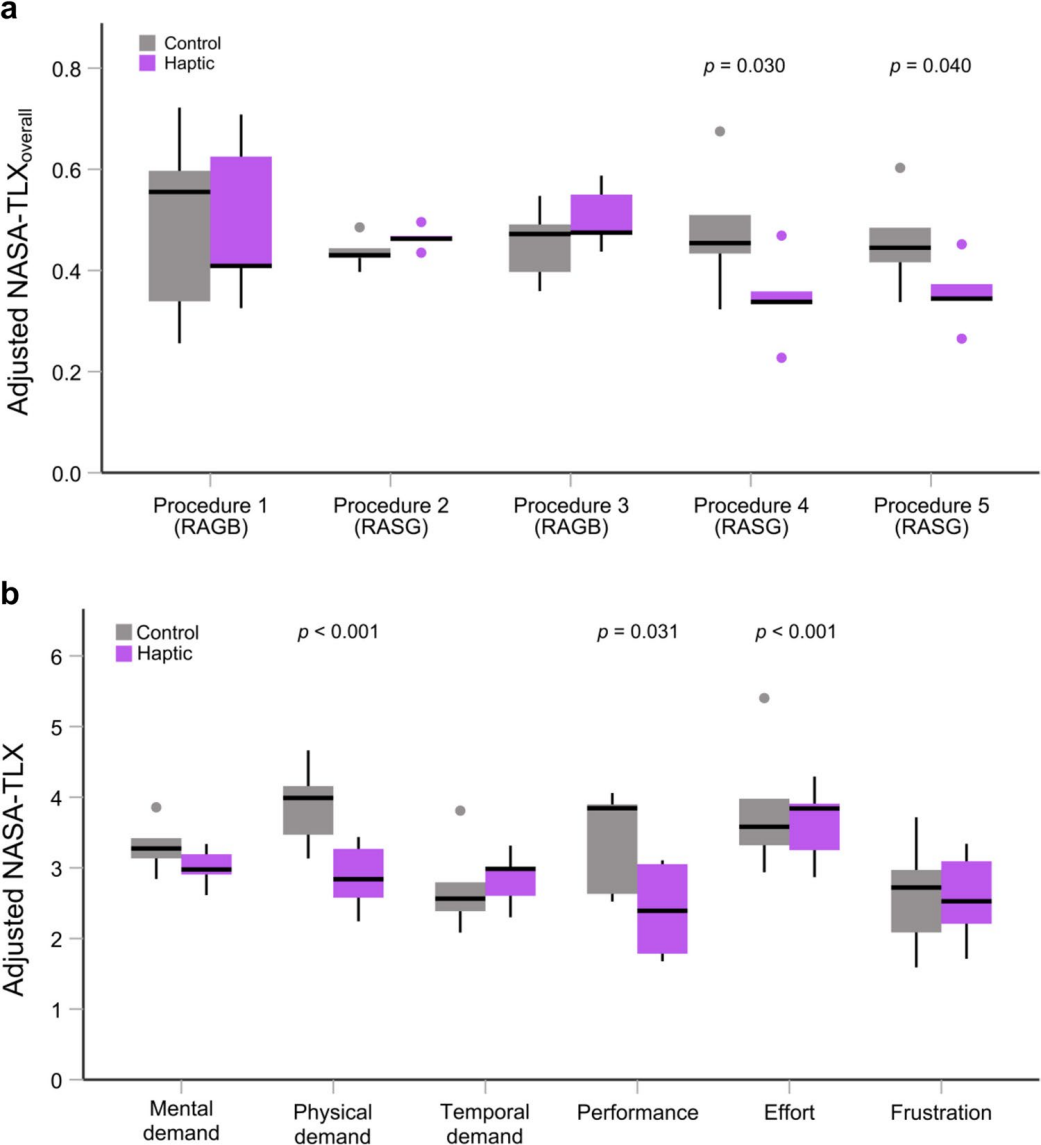
The effect of haptic feedback on *NASA-TLX*_*overall*_ and its subscales during simulation procedures. The thick black line shows the median, the box shows the inter-quartile range (IQR), the thin lines show the range up to 1.5 times the IQR, and dots show outliers. (**a**) *NASA-TLX*_*overall*_ for the five simulated procedures (*n* = 60). The differences between the haptic group and the control group are significant for the fourth and fifth procedures. Significance remained after Bonferroni-Holm correction. (**b**) Analysis of the six NASA-TLX subscales showed significant differences between the groups in physical demand, performance, and effort. For these analyses, *NASA-TLX*_*overall*_ and its subscales scores were adjusted for PGY level and sleep

We found a significant interaction between feedback group and procedure number (*F*(4) = 3.389, *p* = 0.01) and a significant main effect of feedback group (*F*(1) = 6.201, *p* = 0.042). This effect was mainly driven by a general decrease in *NASA-TLX*_*overall*_ (lower workload) for the haptic group, which was especially pronounced at the last two simulated procedures. The haptic group showed a significantly lower *NASA-TLX*_*overall*_ than the control group in both the fourth and fifth procedures (Procedure 4: *p*_*(Holm)*_ = 0.030, Procedure 5: *p*_*(Holm)*_ = 0.040, Table 4, Fig. 4a). However, no significant difference was found in the first three procedures (with* p* > 0.4). The main effect of procedure number was not significant (*F*(4) = 1.113, *p* = 0.370), indicating that the average workload did not differ across the five simulated procedures.

**Table 4:** Comparison of *NASA-TLX*_*overall*_ by feedback group

Setting	Procedure number	Procedure	Haptic	Control	*p*-value
Simulation^a^ *(RAGB, n* = *24* *RASG, n* = *36)*	1	RAGB	0.493 ± 0.049	0.512 ± 0.049	0.859
	2	RASG	0.454 ± 0.047	0.444 ± 0.042	0.887
	3	RAGB	0.500 ± 0.030	0.460 ± 0.027	0.405
	4	RASG	0.304 ± 0.035	0.510 ± 0.031	**0.030**
	5	RASG	0.327 ± 0.029	0.478 ± 0.025	**0.040**
Live OR^b^ *(n* = *36)*	1	RASG	0.475 ± 0.044	0.566 ± 0.040	0.164
	2	RASG	0.557 ± 0.052	0.532 ± 0.047	0.727
	3	RASG	0.498 ± 0.058	0.508 ± 0.053	0.905

Data presented are mean ± SEM. The *p-*values show ANCOVA between-group differences

NASA-TLXoverall for the simulation setting^a^ is adjusted for PGY level and sleep

NASA-TLXoverall for the live OR setting^b^ is adjusted for PGY level

*p-*values < 0.05 are considered statistically significant and are marked in bold

RAGB: robotic-assisted gastric banding, RASG: Robotic-assisted sleeve gastrectomy, OR Operating room

When assessing the effect of feedback group on the six individual NASA-TLX subscales (Haptic: *n* = 30, Control: *n* = 30), the haptic group had a significantly lower scores of physical demand (2.77 ± 0.204 vs. 3.99 ± 0.20, *F*(1) = 14.436, *p* < 0.001), higher levels of satisfaction regarding their performance (2.46 ± 0.27 vs. 3.41 ± 0.27, *F*(1) = 4.896, *p* = 0.031, and lower effort required (3.18 ± 0.17 vs. 4.29 ± 0.17, *F*(1) = 17.012, *p* < 0.001), Fig. 4b) compared to the control group. However, the differences in mental demand, temporal demand, and frustration were not significant (all had *p* > 0.19).

### Live OR setting

For all 76 live RASG cases, an independent t test (Haptic: *n* = 45, Control: *n* = 31) was performed to evaluate the effect of haptic feedback in the live OR setting. The haptic group showed significantly lower *NASA-TLX*_*overall*_ compared to the control group (0.45 ± 0.14 vs. 0.54 ± 0.14, *t*(74) = 2.851, *p* = 0.006).

**Fig. 5:**
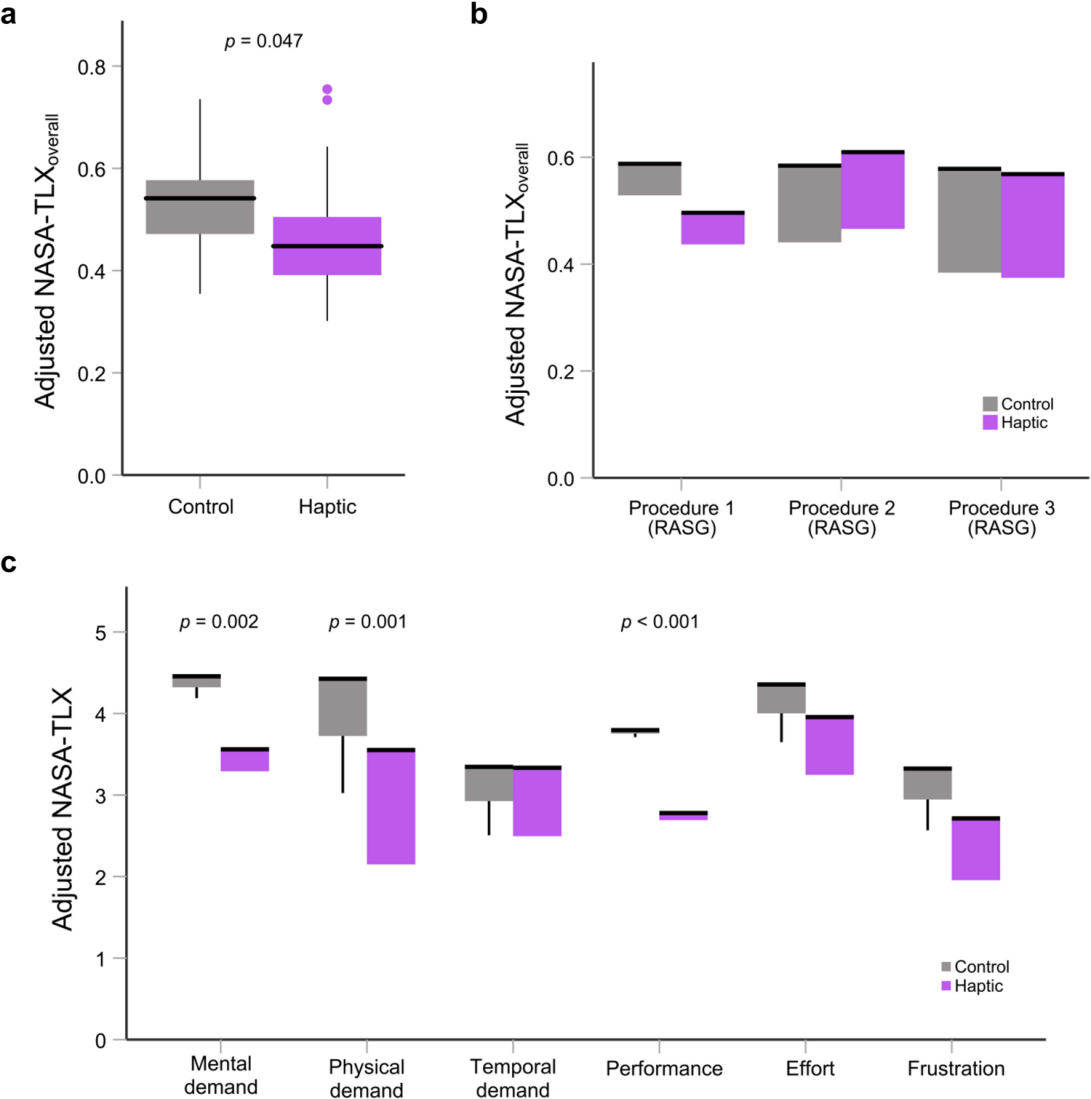
The effect of haptic feedback on *NASA-TLX*_*overall*_ and its subscales in the live OR setting. The thick black line shows the median, the box shows the inter-quartile range (IQR), the thin lines show the range up to 1.5 times the IQR, and dots show outliers. *NASA-TLX*_*overall*_ showed **a** lower workload for the haptic group across 76 live RASG cases (Haptic: *n* = 45, Control: *n* = 31) and **b** no difference in the first three RASG live procedures (*n* = 36). **c** Analysis of the NASA-TLX workload subscales showed a significantly lower mental demand, lower physical demand, and better perceived performance in the haptic group. Here, the *NASA-TLX*_*overall*_ and its subscales were adjusted for PGY level

A two-sample Wilcoxon rank-sum test showed that the difference in OR case experience between groups was not significant (*p* = 0.374). Nonetheless, given the imbalanced number of cases performed by the two groups in the live OR, we also include the total number of cases completed as a covariate alongside PGY level. ANCOVA revealed a significant difference in the *NASA-TLX*_*overall*_ (*F*(1) = 4.085, *p* = 0.047) with a lower workload for the group of residents that received haptic feedback during training compared to the control group (0.46 ± 0.02 vs. 0.52 ± 0.02, Fig. 5a).

Over the course of their bariatric surgery rotations, individual participants performed between 3 and 14 total live RASG procedures after the initial simulated procedures. To consider a balanced number of cases for each group, a mixed ANCOVA was performed on the *NASA-TLX*_*overall*_ for only the first three live procedures (*n* = 36) using PGY level as the covariate. This analysis revealed no significant main effect of group (*F*(1) = 0.165, *p* = 0.696, Fig. 5b) and no interaction between group and procedure (*F*(2) = 2.345, *p* = 0.128). There was also no significant main effect of procedure number (*F*(2) = 1.257, *p* = 0.311).

From the individual NASA-TLX scores across all procedures (Haptic: *n* = 45, Control: *n* = 31), the haptic group reported a significantly lower mental demand (3.49 ± 0.17 vs. 4.39 ± 0.21, *F*(1) = 10.804, *p* = 0.002), lower physical demand (3.18 ± 0.17 vs. 4.06 ± 0.20, *F*(1) = 11.201, *p* = 0.001), and also lower workload due to self-rated performance (corresponding with a better self-perceived performance) (2.76 ± 0.16 vs. 3.77 ± 0.20, *F*(1) = 15.843, *p* < 0.001, Fig. 5c) compared to the control group in the OR. There was a trend toward lower frustration for the haptic group (*p* = 0.091); however, no significant differences were found for temporal demand or effort (*p* > 0.20 for both).

## Discussion

Robotic surgery has gained increasing acceptance [4, 42] since it was first reported in 1988 [43], and it may confer advantages over laparoscopic surgery in select procedures [2, 44, 45]. Prior research has shown that cognitive overload can affect the performance of surgeons and adversely affect patient safety [46, 47], so workload is considered an important non-technical aspect influencing surgical performance [10, 48]. This study examined the relationship between residents’ subjective workload and their level of experience, procedure completion time, and baseline stress. Furthermore, this study shed light on the effects of haptic feedback of instrument vibrations during procedure-specific dry-lab training on the subsequent workload experienced by surgical trainees during live robot-assisted bariatric surgery.

Our findings suggest that incorporation of naturalistic vibrotactile haptic feedback to robotic bariatric surgical training could significantly reduce perceived workload during real bariatric surgery. The overall workload reported over the five simulated procedures was strongly associated with whether the participant had haptic feedback. Even though haptic feedback was provided during only the first four simulated procedures and was removed for the final procedure (Fig. 2), the overall workload of the residents in the haptic group reduced significantly after the third procedure. The reduction in overall workload persisted after controlling for procedure completion time and baseline STAI score. In contrast, the overall workload increased from the baseline to the second procedure and slightly decreased in the third procedure for the control group. Thus, haptic feedback of instrument vibrations during surgical skill acquisition may reduce perceived workload independently from operative time and baseline STAI score. The reduced overall workload reported by the haptic group suggests that training with haptic feedback is associated with reduced cognitive burden while learning to operate a surgical robot after controlling for other factors. Furthermore, reduced physical demand, increased perceived performance, and reduced effort in the individual subscales of the NASA-TLX may demonstrate the value of haptic feedback to accelerate learning of robotic surgery.

This study is novel in its tracking of workload from the simulated to live OR settings with a focus on a change to the sensory input received by surgeons. Haptic feedback during pre-clinical simulation training is associated with lower workload experienced by trainees during real surgery even though these live procedures were performed without any haptic feedback. Even after controlling for a resident’s PGY level (representing their overall prior experience) and the number of procedures they had already completed in the study (representing their direct operative experience), the reduction in their workload persisted. The claim that improved visualization can compensate for reduced haptic feedback was studied post facto after regulatory approval of the da Vinci Surgical System, and it is essential to note that these studies consistently demonstrate that experienced surgeons can compensate for this sensory deficit more effectively than trainees [49–51]. Our findings indicate that practicing with haptic feedback has a significant effect on workload regardless of the resident’s experience level. This somewhat contradicts the findings from Cundy et al. [51] that surgeon experience compensates for the absence of haptic feedback during suturing in robotic surgery, though our study included only surgeons in training and not attending surgeons. This study and prior studies support the claim that there exists a learning curve to operating using vision to compensate for the sense of touch. As with any surgical procedure, there is a potential for increased errors and complications early in a learning curve. The surgical profession is reputable for the high mental and physical demands experienced by its members, so the findings of this study apropos to the reduction of workload during surgical learning are relevant to the quality of life of surgical trainees in addition to the safety of patients.

Moreover, our analysis of the NASA-TLX subscales indicates that physical demand and perceived performance in both simulated and live OR settings were positively affected by the addition of haptic feedback during simulation. This finding suggests that providing vibrotactile haptic feedback in the simulation setting alone may reduce trainee workload in the live OR. However, we found no effect of haptic feedback nor any interaction between group and procedure in the more detailed analyses of the first three live OR procedures, potentially because early clinical experience varies widely, and/or because this comparison is underpowered. The better performance perceived by participants could be due to the additional sensory information provided by the haptic feedback of instrument vibrations, which gave the participants in the haptic group a more natural sense of control of the instruments; the control group had no access to such sensory information. Consequently, we speculate that providing vibrotactile haptic feedback during training could enhance resident learning in advance of real operations and accelerate development of the ability to compensate for loss of all forms of haptic feedback using visual cues.

Cognitive demand is said to be affected not only by primary tasks but also by factors like well-being, fatigue, and experience [52]; this statement matches what we found from the regression analysis, namely that baseline stress level, hours of sleep, and overall prior experience play significant roles in the perceived workload. Our findings indicate that direct operative experience (represented by the total number of cases a resident had performed in the study) influences participant workload in the live OR setting but not in the simulation setting, potentially because the OR is a more high-stakes environment [11]. It is notable that the difference of reported workload by a PGY-3 resident compared to a PGY-7 resident was quantified as approximately 10% of the maximum reportable NASA-TLX score based on the results of this study. This value is suggested by the coefficients of PGY level in the regression equations for predicting *NASA-TLX*_*overall*_, and it appeared to be preserved in both the simulated and OR settings. To our knowledge, this study is novel in the quantification of the contribution of surgical experience on workload and demonstration of its consistency between the simulated and OR settings.

To our knowledge, this is also the first study to assess the impact of haptic feedback on subjective workload during resident training in both simulation and the real OR. However, with only six participants in each group, there is a strong possibility that the study is inadequately powered. The initial power calculation of this study had anticipated twice as many participants, but changes in the bariatric surgical rotation structure after study initiation precluded the anticipated participant recruitment. Furthermore, the workload evaluation was performed over a relatively short time window compared to other work that has reported the learning curve of robotic-assisted sleeve gastrectomy to be about 20 cases [53]. It would be beneficial to track OR performance for longer than three procedures in the future, though the variation in experienced surgical volume by trainees and even attending physicians can make such a study challenging to implement.

Since the studied bariatric procedure (RASG) is a commonly performed and relatively lower difficulty operation in routine situations, the applicability of this study’s findings to more difficult robotic surgical procedures such as oncologic resections is subject to further investigation. Additionally, this study was focused on the effects of vibrotactile feedback on resident learning, which will become increasingly relevant as robotic surgical training is integrated into surgery residency programs. The relative simplicity of the RASG compared to other robotic surgeries and even other foregut surgeries made it a safe operation for residents to perform at the console and thus a safe operation for this study. Other works have shown that haptic feedback may be more helpful as the complexity of a task increases [54, 55]. Unfortunately, we relied on self-report and thus were not able to precisely quantify our participants’ prior experience with relevant forms of surgery. However, it was found that the number of prior laparoscopic experience did not differ significantly between groups. Finally, it is a limitation that the simulation proctor could not be blinded to subject identity or feedback condition. It is thus possible that the proctor was biased toward providing better bedside assistance during the simulation session to residents receiving haptic feedback; however, it seems relatively unlikely for an unconscious bias to produce such significant differences in perceived workload scores and perceived performance.

## Conclusions

Mental overload can have a detrimental impact on surgical performance. Providing haptic feedback of instrument vibrations during surgical training may enable residents to perform real robotic bariatric surgery with reduced workload and enhance their experience during real robotic surgery. Furthermore, assuming that an individual has a limited amount of cognitive reserve, it is worthwhile to pursue measures that reduce workload due to novelty of a surgical device so that mental resources can be focused on the performance of the surgical procedure and handling any challenges that arise. This study’s findings indicate that it may be beneficial to utilize haptic feedback of instrument vibrations for training residents in robotic surgery. It is essential to note that feeling mechanical vibrations is only one modality of the sense of touch, with light touch, forces, torques, contact location, and temperature being additional components of the sense of touch that remain opportunities for additional study. At present, we cannot anticipate how well our results would transfer to other forms of haptic feedback, including those becoming available in current clinical robots.

While the value of haptic feedback may remain controversial as robotic surgery becomes more widely practiced and additional robotic surgical systems come to market, this study provides evidence that haptic feedback is beneficial even under the improved visualization available in robotic surgery, at least in the context of early learning. Further larger-scale investigations using objective evaluation of workload as well as surgical task performance are required to provide us with an even better understanding of how workload and operative experience are affected by this form of haptic feedback. Given that all open surgeries and nearly all non-teleoperated tasks performed by humans are performed under conditions where the sense of touch is entirely present, it is essential to understand the challenges of learning to operate without the natural sense of touch, particularly if subsequent robotic surgical systems continue to lack complete and robust systems for haptic feedback.
